# Safety, Efficacy, Pharmacokinetic and Pharmacodynamic evaluation of YF-H-2015005 for mobilizing Hematopoietic stem cells in Non-Hodgkin's Lymphoma Patients

**DOI:** 10.7150/jca.48748

**Published:** 2020-07-25

**Authors:** Weiping Liu, Yan Xie, Lingyan Ping, Min Jiang, Guanmin Zhang, Yimin Cui, Junyu Xu, Meng Wu, Xin Leng, Xiaopei Wang, Shufang Wang, Jun Zhu, Yuqin Song

**Affiliations:** 1 Key Laboratory of Carcinogenesis and Translational Research (Ministry of Education), Department of Lymphoma, Peking University Cancer Hospital & Institute, No.52 Fucheng Road, Haidian District, Beijing 100142, China.; 2 Key Laboratory of Carcinogenesis and Translational Research (Ministry of Education), National Drug Clinical Trial Center (GCP Center), Peking University Cancer Hospital & Institute, No.52 Fucheng Road, Haidian District, Beijing 100142, China.; 3 Key Laboratory of Carcinogenesis and Translational Research (Ministry of Education), Department of Pharmacy, Peking University Cancer Hospital & Institute, No.52 Fucheng Road, Haidian District, Beijing 100142, China.; 4 Peking University First Hospital, Department of Pharmacy, No.8 Xishiku Street, Xicheng District, Beijing 100034, China.; 5 Hefei Yifan Biopharmaceuticals Inc., Intersection between Jinxiu avenue and qinglongtan road, Hefei 610000, China.

**Keywords:** Hematopoietic Stem Cell Mobilization, Drug Evaluation, Pharmacokinetics, Safety, Lymphoma, Non-Hodgkin's

## Abstract

**Background:** Targeting the interaction between SDF1 and CXCR4 may provide an opportunity to intervene in the hematopoietic stem cell mobilization process.

**Aim:** The present study aimed to investigate the safety, efficacy, pharmacokinetic and pharmacodynamic profiles of YF-H-2015005, a CXCR4 antagonist, for the mobilization of hematopoietic stem cells (HSCs). **Methods:** A total of 15 patients with non-Hodgkin's lymphoma (NHL) eligible for autologous hematopoietic stem cell transplantation were enrolled. All patients achieved a partial or complete remission after the first- or second-line therapy. Granulocyte colony stimulating factor (G-CSF) was given in the morning for 8 consecutive days, and 0.24 mg/kg YF-H-2015005 was subcutaneously administered in the evening of the 4^th^ day of G-CSF treatment for up to four days. Apheresis was performed 9-10 hours following each dose of YF-H-2015005. Results: YF-H-2015005 was rapidly absorbed and eliminated, with T_max_ and t_1/2_ of 0.5 and 5.04 ± 1.00 hours, respectively. Moreover, the mean peripheral blood CD34^+^ cell counts were elevated by 2.0- to 2.9-fold from 2 to 24 hours, and reached the maximum level of 76.5 ± 53.9 cells/kg at 10 hours after YF-H-2015005 treatment. Fourteen (93%) out of 15 NHL patients achieved a minimum target of ≥2×10^6^/kg CD34^+^ cells. Furthermore, there was no grades 3-4 treatment-related adverse event observed among these patients. Conclusion: YF-H-2015005 can serve as a safe, effective agent in combination with G-CSF for CD34^+^ hematopoietic progenitor cell mobilization in NHL patients.

## Introduction

The interaction of CXC chemokine receptor 4 (CXCR4) and its ligand CXCL12 (also referred as stromal cell-derived factor-1, SDF-1) plays an essential role in regulating the mobilization of hematopoietic stem cells (HSCs) 1,2. SDF-1 is constitutively secreted in bone marrow stromal cells, while the glycoprotein-coupled receptor CXCR4 with SDF-1 homogeneity is mainly generated by HSCs 3,4. Therefore, targeting the interaction between SDF1 and CXCR4 may provide an opportunity to intervene the mobilization of HSCs. Plerixafor, a CXCR4 antagonist, is able to enhance the steady-state release of SDF-1 into the bloodstream of both mice and non-human primates 5, and can lead to a 3- to 6-fold elevation in the absolute numbers of peripheral blood (PB) CD34^+^ cells in healthy volunteers 6. It is worth noting that plerixafor can exhibit high effectiveness on the mobilization of HSCs in patients with non-Hodgkin's lymphoma (NHL) or multiple myeloma 7. In 2008, plerixafor was approved by the U.S. Food and Drug Administration for the mobilization of HSCs to the PB for collection and subsequent autologous hematopoietic stem cell transplantation (HSCT) 8.

**Figure 1:**
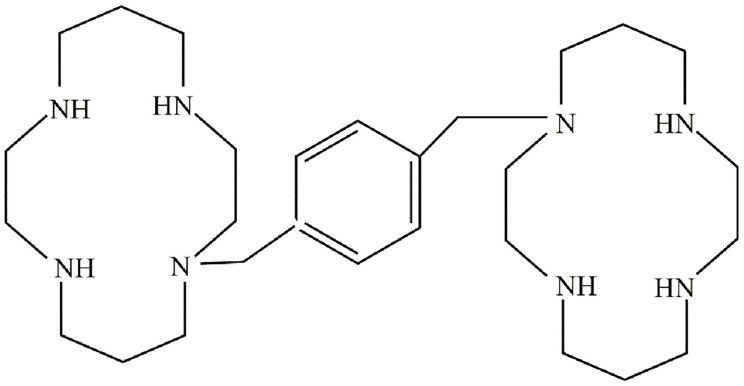
Structural formula of YF-H-2015005.

YF-H-2015005 (l,1'-[1,4-phenylenebis(methylene)]-bis-1,4,8,11-tetraazacyclotetradecane) was a CXCR4 antagonist developed by Hefei Yifan Biopharmaceuticals Inc. (Anhui, China). It displayed the structural formula C28H54N8, with a molecular mass of 502.79 g/mol. The molecular structure of YF-H-2015005 was illustrated in **Figure 1.** Preclinical trials have demonstrated that YF-H-2015005 is effective and safe for enhancing the mobilization of HSCs. In this study, we determined the efficacy, safety, pharmacokinetic (PK) and pharmacodynamics (PD) profiles of YF-H-2015005 in combination with granulocyte colony stimulating factor (G-CSF) for CD34^+^ HSC mobilization in NHL patients. Furthermore, the enhancement effect of YF-H-2015005 on HSC mobilization in NHL patients was also evaluated.

## Materials and Methods

### Study design

The ethical approval for this open-label, single arm study was obtained from the Ethics Committee of the Peking University Cancer Hospital and Institute (Beijing, China), and all procedures were conducted in accordance with the Declaration of Helsinki. All participants provided written informed consent prior to study enrollment. This study was registered on www.chinadrugtrials.org.cn (registration number: CTR20170925).

The inclusion criteria of this study were: (i) NHL patients eligible for autologous HSCT, males and females, aged 18-65 years; (ii) achieving a partial or complete remission after the first- or second-line therapy; (iii) an Eastern Cooperative Oncology Group (ECOG) performance status score of 0 or 1; (iv) did not overlap with the toxicity of other chemotherapeutic or anti-cancer drugs; (v) an actual body weight less than 175% of ideal body weight; (vi) negative for bone marrow involvement within 45 days prior to enrollment; and (vii) negative for human immunodeficiency and hepatitis viruses.

The main exclusion criteria were as follows: (i) chronic lymphocytic leukemia; (ii) failed previous HSC collections; (iii) history of allogeneic or autologous HSCT; (iv) history of pelvic radiotherapy; and (v) secondary central nervous system involvement. In addition, NHL patients with creatinine clearance rate <50 mL/min as well as abnormal liver function test results (>2.5 times upper limit of normal) were excluded.

### Combination treatment of G-CSF and YF-H-2015005

All patients were given a daily subcutaneous dose of G-CSF (10 µg/kg/day) each morning for 8 consecutive days. YF-H-2015005 with a dose of 0.24 mg/kg was subcutaneously initiated on the evening of the 4^th^ day of G-CSF treatment and continued for up to 4 days. Apheresis was performed 9-10 hours following evening dose of YF-H-2015005. This process was continued daily for up to 4 days or until ≥2×10^6^/kg CD34^+^ cells were collected.

### PK and PD evaluation

For PK analysis, blood samples were collected immediately before starting YF-H-2015005 treatment, and consecutively at 0.25, 0.5, 1, 2, 4, 6, 8, 10, 16 and 24 hours following its administration and prior to apheresis. HSC apheresis was carried out using a COM.TEC instrument (Fresenius Kabi, Germany). Then, 4 mL of peripheral venous blood sample was collected into the heparin sodium tube at each time point of PK sampling. The tube was inverted 5 times and immediately placed on ice. After centrifuging at 3000 rpm for 10 minutes, the resulting plasma was kept at -70°C. The plasma from each sample was split into two approximately equal aliquots.

All samples were evaluated by liquid chromatography coupled with tandem mass spectrometry (LC-MS/MS). The linear range of the assay was 2 to 1000 ng/mL. The precision values of the quality control samples (6, 40, 400 and 750 ng/mL) were all ≤8.5%, with a bias of -2.7~13.4%. The PK parameters analyzed included the area under the curve from time 0 to t (AUC_0-t_), area under the curve from time 0 to infinity (AUC_0-∞_), maximum observed concentration (C_max_), time to achieve C_max_ (T_max_), apparent plasma clearance (CL), apparent volume of distribution (V_z_), and apparent terminal elimination half-life (t_1/2_). When multiple doses of YF-H-2015005 were administered, PK parameters were consisted of the steady-state trough concentration (C_ssmin_), steady-state peak concentration (C_ssmax_), average steady-state plasma drug concentration (Cav), steady-state area under the curve (AUC_ss0-24_), coefficient of fluctuation (Fl), and accumulation ratio (Ra). The values for all PK parameters were calculated with non-compartmental analysis using WinNonlin Professional version 6.3 software (Pharsight Corp, Mountain View, CA, USA).

For PD analysis, blood samples were collected immediately prior to dosing with YF-H-2015005 and then at 2, 4, 6, 8, 10, 16, and 24 hours after its administration. Four milliliter sample of peripheral venous blood was collected into the heparin sodium tube at each time point. The absolute number and percentage of CD34 cells were assessed by fluorescence-activated cell sorting method using a Beckman-Coulter FC500 flow cytometer and appropriate reagents.

### Safety and efficacy assessments

The safety profiles were evaluated by clinical and laboratory parameters, including adverse event (AE), serious AE (SAE), treatment-related AE, treatment-related SAE, and the AEs were monitored throughout the entire study period. All the clinical signs and symptoms were recorded, and a further laboratory examination or electrocardiogram was carried out (as dictated by the occurrence of AE).

The efficacy endpoint was the proportion of NHL patients treated with YF-H-2015005 who achieved a minimum collection target of ≥2×10^6^/kg CD34^+^ cells (actual body weight) within 4 days of apheresis.

### Statistical analysis

All statistical analyses were conducted using IBM SPSS Statistics for Windows, Version 21.0 (IBM Corp., Armonk, New York, USA). Continuous variables were determined using the Student's t-test. Categorical variables were analyzed using the Pearson's chi-squared test or Fisher's exact test. All tests applied were two-tailed, and a *p*-value of less than 0.05 was deemed to be statistically significant.

## Results

### Baseline characteristics

In this study, 15 NHL patients (11 males and 4 females) were recruited, and none of them received fludrarabine or lenalidomide before HSC mobilization. The median age was 51 years. About 67% of patients had mature B-cell lymphoma. The median cycles of prior chemotherapy was 6. None received radiotherapy. **Table 1** revealed the demographic and disease characteristics of the enrolled NHL patients.

### PK and PD profiles of YF-H-2015005

**Figure 2:**
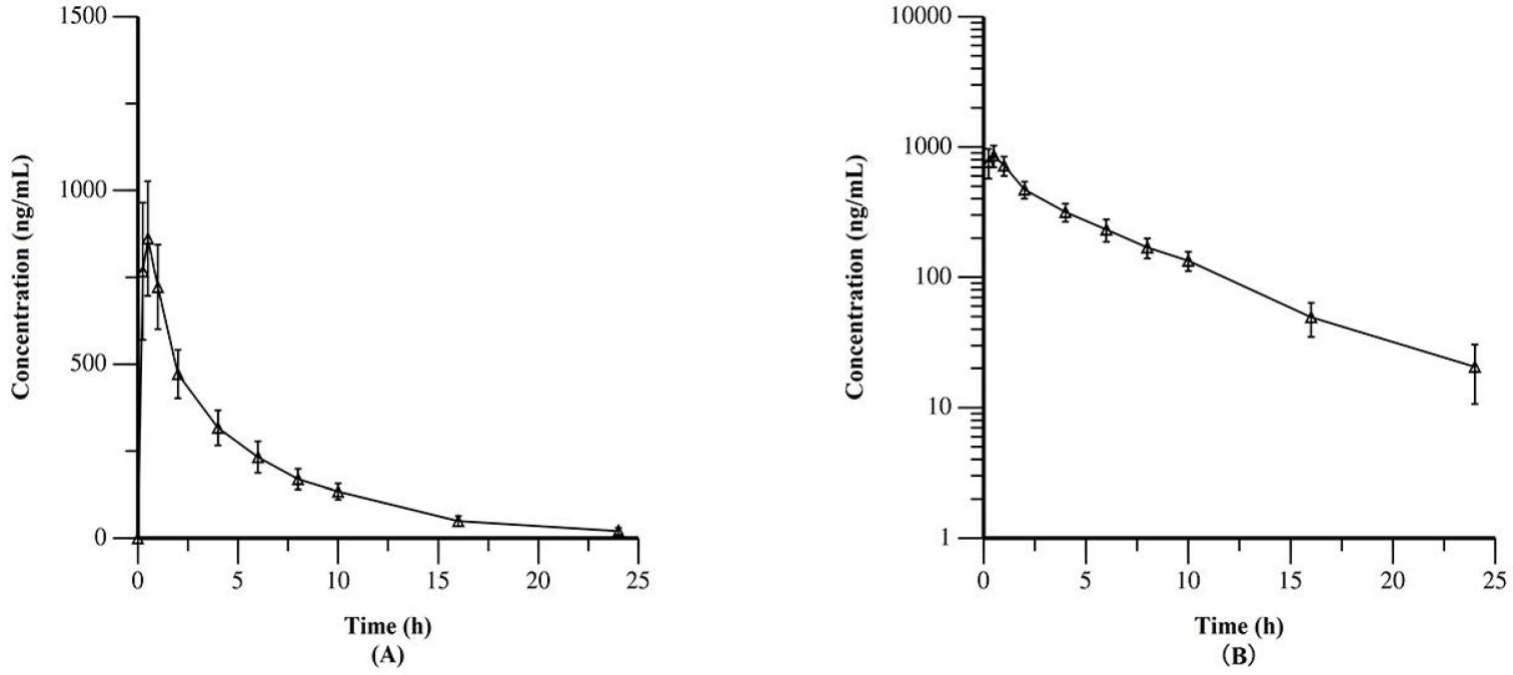
Mean plasma concentrations of YF-H-2015005 over time as expressed on linear (A) and semilogarithmic (B) scales.

**Table 2** summarized the PK data of YF-H-2015005. The results demonstrated that YF-H-2015005 was rapidly absorbed in all 15 patients without a lag time after its first administration. Moreover, its median values of Tmax and median biological half-life (t_1/2_) were 0.5 and 5.04 hours, respectively. **Figure 2** showed the mean plasma concentrations of YF-H-2015005 over time on both linear and semilogarithmic scales. Only one patient received all 4 consecutive doses of YF-H-2015005 as stipulated in the study protocol, and hence the PK parameters of steady state were calculated based on this patient (**Appendix Table S1**). After administrating multiple doses of YF-H-2015005, the C_ssmin_, C_ssmax_, T_max_, t_1/2_, steady-state AUC_ss0-24_, F1 and Ra of this patient were 21.6 ng/mL, 602 ng/mL, 0.5 hours, 5.83 hours, 3210 h· ng/mL, 433.9% and 1.06, respectively.

**Table 3** summarized the PD characteristics of YF-H-2015005 in NHL patients. Following YF-H-2015005 treatment, the mean PB CD34^+^ cell counts increased by 2.0- to 2.9-fold over baseline from 2 to 24 hours, and reached the maximum level of 76.5 ± 53.9 cells/μL at 10 hours after dosing. As mentioned above, only 1 patient received 4 doses of YF-H-2015005. The PD characteristics of YF-H-2015005 in this patient was summarized in Appendix Table S2.

### Efficacy of YF-H-2015005 for HSC mobilization

Fourteen (93%) patients met the endpoint of ≥2×10^6^/kg CD 34^+^ cells being collected after a median of 1.0 day of apheresis (range: 1-2 days). The median amount of CD34^+^ cells collected was 3.3×10^6^ cells/kg (range: 1.5-10.5×10^6^ cells/kg). The target collection was obtained from 10 patients after only one apheresis session and from 4 patients after two apheresis sessions. Among those patients, 4 (27%) patients achieved the maximal CD34^+^ cell level of ≥5×10^6^ cells/kg. The remaining 1 patient yielded 1.5×10^6^ cells/kg following 4 days of apheresis.

### Safety of YF-H-2015005

All patients had at least one AE. As shown in **Table 4**, treatment-related AEs were observed in 13 (87%) patients, and all of them were grade 1-2. The most frequently reported treatment-related AEs were increased lactate dehydrogenase (67%), followed by hot flashes (13%), diarrhea (7%), insomnia (7%), and hypoglycemia (7%). However, no treatment-related SAEs were observed.

## Discussion

Autologous HSCT is the standard therapeutic modality for patients with NHL 9-12. The successful mobilization of PB stem cells and adequate stem cell collection are of critical importance, as the infused CD34^+^ cell dose can affect the engraftment of platelet and neutrophils 13. The optimal amount of CD34^+^ cells that causes rapid and sustained recovery is determined to be >5×10^6^ cells/kg, while the minimum threshold amount is deemed as >2×10^6^ cells/kg 14. However, failure to achieve sufficient HSC mobilization represents a great challenge in clinical practice, especially for supporting subsequent high-dose therapy and autologous HSCT 15. In a retrospective study 16 conducted on 1,775 patients who underwent stem cell mobilization, 47% of the patients had a suboptimal stem cell collection (CD34^+^ cell level <5×10^6^ cells/kg), among which, 25% had a low collection (2-5×10^6^ cells/kg), 10% had a poor collection (<2×10^6^ cells/kg), and 12% failed collection. Hence, there remains an urgent need to develop more effective, safer, and better tolerated treatment regimens for the mobilization of HSCs.

The greater effectiveness of using plerixafor in combination with G-CSF compared to G-CSF treatment alone has been demonstrated by a double-blind, randomized, placebo-controlled study 17. A total of 298 NHL patients were allocated to receive G-CSF in combination with plerixafor or a placebo. The proportion of NHL patients who achieved the primary endpoint (≥5×10^6^ CD34^+^ cells/kg) was higher in plerixafor group (59%) than in placebo group (20%). It has been reported that plerixafor exhibits similar efficacy in a Chinese population 18. In this randomized, double-blind study involving 101 patients with NHL, the number of patients who achieved a target collection of >5×10^6^ CD34^+^ cells/kg (62% vs. 20%) or >2×10^6^ CD34^+^ cells/kg (88% versus 66%) and underwent autologous transplantation (88% versus 68%) was higher in the plerixafor arm when compared to those in the placebo arm. Based on these prospective randomized studies, pre-emptive use of plerixafor for those patients at risk for HSC mobilization failure has been recommended in clinical practice guidelines 14,15,19.

In the present study, the PK and PD of YF-H-2015005 in patients with NHL were evaluated. The PK findings revealed that YF-H-2015005 was rapidly absorbed and eliminated, with a mean t_1/2_ of 5.04 hours. Moreover, the numbers of CD34^+^ cells in PB samples increased significantly after YF-H-2015005 administration. Treatment with YF-H-2015005 could result in a mean 2.9-fold elevation of PB CD34^+^ cells within 10 hours following YF-H-2015005 administration, by which the mean absolute PB CD34^+^ cells elevated from 27.2 to 76.5 cells/μL. Moreover, 14 (93%) patients achieved the endpoint for CD34^+^ cell collection (≥2×10^6^ cells/kg), with a median CD34^+^ cell count of 3.35×10^6^ cells/kg. These findings of PK and PD data supported the use of YF-H-2015005 dosing regimen and determined the optimal timing of apheresis.

More importantly, the target cell collection was obtained in 67% of patients after a single apheresis session and in 27% of patients after 2 apheresis sessions, which could lead to a reduction in health care costs (e.g., apheresis and hospitalization costs). Only 1 patient did not achieve the minimum target of ≥2×10^6^ CD34^+^ cells/kg required for HSCT; however, similar to the other 14 patients, this patient exhibited a ≥2-fold elevation in PB CD34^+^ cells. In addition, all AEs were mild and transient with no SAEs reported. These findings indicated that YF-H-2015005 is an effective and safe agent for mobilizing HSCs in NHL patients.

In conclusion, our findings clearly demonstrated the potential of YF-H-2015005 as a safe, effective agent for HSC mobilization and subsequent collection by apheresis in patients with NHL. The rapid time course of YF-H-2015005-induced CD34^+^ cell mobilization as well as the relative paucity of adverse effects further supported the clinical applicability of this agent. The magnitude of an increased amount of PB CD34^+^ cells after treatment with 0.24 mg/kg YF-H-2015005 was sufficient to indicate the clinical significance of this agent for mobilizing HSCs.

## Supplementary Material

Supplementary tables.

## Figures and Tables

**Table 1:** Baseline characteristics of 15 patients treated with YF-H-2015005

Characteristics	All patients (%)
**Age, years**	
Mean (SD)	47.8 (10.8)
Median (range)	51.0 (32.0-64.0)
**Ethnicity, n (%)**	
Han	14 (93.3)
Others	1 (6.7)
**Gender, n (%)**	
Male	11 (73.3)
Female	4 (26.7)
**Body weight, kg**	
Mean (SD)	71.3 (13.0)
Median (range)	74.0 (50.0-86.0)
**Pathology type**	
Lymphoblastic lymphoma	1 (6.7)
Peripheral T/NK cell lymphoma	4 (26.7)
ALK-negative ALCL/ PTCL/ AITL	1/1/2
Mature B-cell lymphoma	10 (66.6)
DLBCL/MCL/BL/tFL/ Unclassified	5/2/1/1/1
**Stage**	
I	2 (13.3)
II	5 (33.4)
III	2 (13.3)
IV	6 (40.0)
**Lines of chemotherapy**	
First line	10 (66.7)
Second line	5 (33.3)
**Cycles of chemotherapy**	
Mean (SD)	7 (3)
Median (range)	6 (4-12)
**Radiotherapy**	
Yes	0 (0.0)
No	15 (100.0)
**Disease status**	
Complete remission	11 (73.3)
Partial remission	4 (26.7)

AITL, angioimmunoblastic T-cell lymphoma; ALCL, ALK-, anaplastic cell lymphoma, anaplastic lymphoma kinase negative; BL, Burkitt lymphoma; DLBCL, diffuse large B-cell lymphoma; MCL, mantle cell lymphoma; PTCL NOS, peripheral T-cell lymphoma, not otherwise specified; SD, standard deviation; tFL, transformed follicular lymphoma.

**Table 2:** Values for PK parameters after a single dose of YF-H-2015005 (0.24 mg/kg, s.c.) (n = 15)

PK parameters	Values (Mean ± SD)
t_1/2_, h	5.04 ± 1.00
T_max_, h^*^	0.50 (0.25, 1.00)
C_max_, ng/mL	876 ± 171
AUC_0-10h_, h·ng/mL	3336 ± 481.2
AUC_0-24h_, h·ng/mL	4166 ± 604.3
AUC_0-∞_, h·ng/mL	4328 ± 637.1
V_z_, L/kg	0.4 ± 0.1
CL, L/h/kg	0.06 ± 0.01

*Indicated as the median (minimum, maximum). AUC_0-10h_, area under the curve from time 0 to 10h; AUC_0-24h_, area under the curve from time 0 to 24h; AUC_0-∞_, area under the curve from time 0 to infinity; CL, apparent clearance; C_max_, maximum observed concentration; PK, pharmacokinetic; t1/2, apparent terminal half-life; T_max_, observed maximum concentration; V_z_, apparent volume of distribution.

**Table 3:** Changes in peripheral blood CD34+ cell count after the first dose of YF-H-2015005

Time (hrs)	CD34+ cell count (Mean ± SD, cells/μL)	Fold-increase in CD34+ cell count (Mean ± SD)
0	27.2 ± 21.1	-
2	67.5 ± 44.7	2.6 ± 1.0
4	73.4 ± 46.3	2.8 ± 1.0
6	73.0 ± 47.9	2.8 ± 0.7
8	72.4 ± 46.4	2.8 ± 0.8
10	76.5 ± 53.9	2.9 ± 0.6
16	52.6 ± 30.4	2.2 ± 0.7
24	48.1 ± 25.4	2.0 ± 0.7

SD, standard deviation.

**Table 4:** Summary of safety

	Number (%)
**Adverse event**	15 (100.0)
Treatment-related adverse event	13 (86.7)
Serious adverse event	0 (0.0)
Treatment-related serious adverse event	0 (0.0)
**Common treatment-related adverse events**	
Increased lactate dehydrogenase	10 (66.7)
Hot flashes	2 (13.3)
Diarrhea	1 (6.7)
Insomnia	1 (6.7)
Hyperhidrosis	1 (6.7)

## Abbreviations

AE: adverse event
AUC_0-10h_: area under the curve from time 0 to 10h
AUC_0-24h_: area under the curve from time 0 to 24h
AUC0_-∞_: area under the curve from time 0 to infinity
AUC_SS_: steady-state area under the curve
C_av_: average steady-state plasma drug concentration
CL: apparent clearance
C_max_: maximum observed concentration
C_ss.max_: steady-state peak concentration
C_ss,min_: steady-state trough concentration
CXCR4: CXC chemokine receptor 4
ECOG: Eastern Cooperative Oncology Group
Fl: coefficient of fluctuation
G-CSF: granulocyte colony stimulating factor
HSCs: hematopoietic stem cells
NHL: non-Hodgkin's lymphoma
PB: peripheral blood
PD: pharmacodynamics
PK: pharmacokinetic
SDF-1: stromal cell-derived factor-1
Ra: accumulation ratio
SAE: serious AE
t1/2: apparent terminal half-life
T_max_: observed maximum concentration
V_z_: apparent volume of distribution
