# Supplementary material for: Safety, Efficacy, Pharmacokinetic and Pharmacodynamic evaluation of YF-H-2015005 for mobilizing Hematopoietic stem cells in Non-Hodgkin's Lymphoma Patients
Source: J Cancer. 2020 Jul 25;11(19):5635–40. doi: 10.7150/jca.48748 (PMC7477452; doi:10.7150/jca.48748)
Supplement: Supplementary file 1 — Supplementary tables. [file jcav11p5635s1.pdf]

Table S1. PK parameters after multiple doses of YF-H-2015005 (n=1)

PK parameters	Values	
	Day 1 (Single)	Day 4 (Repeat)
$t_{1/2}$, h	5.42	5.83
T_{max}, h	0.50	0.50
C_{max}, ng/mL	714	-
$C_{ss.max}$	-	602
$C_{ss,min}$	-	21.6
C_{av}	-	134
$AUC_{0\text{-}10h}$, h·ng/mL	2549	2486
$AUC_{0\text{-}24h}$, h·ng/mL	3162	-
$AUC_{0\text{-}\infty}$, h·ng/mL	3284	-
AUC_{SS}, h·ng/mL	-	3210
V_z, L/kg	0.6	-
CL, L/h/kg	0.07	-
Ra	-	1.06
Fl, %	-	433.9

$AUC_{0\text{-}10h}$, area under the curve from time 0 to 10h; $AUC_{0\text{-}24h}$, area under the curve from time 0 to 24h; $AUC_{0\text{-}\infty}$, area under the curve from time 0 to infinity; AUC_{SS}, steady-state area under the curve; C_{av}, average steady-state plasma drug concentration; CL, apparent clearance; C_{max}, maximum observed concentration; $C_{ss.max}$, steady-state peak concentration; $C_{ss,min}$, steady-state trough concentration; Fl, coefficient of fluctuation; PK, pharmacokinetic; Ra, accumulation ratio; t1/2, apparent terminal half-life; T_{max}, observed maximum concentration; V_z, apparent volume of distribution

Table S2. Changes in peripheral blood CD34$^+$ cells following multiple doses of YF-H-2015005 (n=1)

	Day 1 (Single)		Day 4 (Repeat)	
Time (h)	CD34$^+$ cell count (cells/μL)	Fold-increase in CD34$^+$ cells	CD34$^+$ cell count (cells/μL)	Fold-increase in CD34$^+$ cells
0	5.0	-	6.0	-
2	4.0	0.8	9.0	1.5
4	3.3	0.7	5.0	0.8
6	9.3	1.9	5.0	0.8
8	4.0	0.8	4.0	0.7
10	9.8	2.0	4.0	0.7
16	11.0	2.2	5.0	0.8
24	6.0	1.2	5.0	0.8
